# The Relationship Between Cervical Column Curvature and Sagittal Position of the Jaws: Using a New Method for Evaluating Curvature

**DOI:** 10.5812/kmp.iranjradiol.17351065.3379

**Published:** 2011-11-25

**Authors:** Tahereh Hosseinzadeh Nik, Pejman Janbaz Aciyabar

**Affiliations:** 1 Department of Orthodontics, Faculty of Dentistry and Dental Research Center, Tehran University of Medical Sciences, Tehran, Iran; 2 Private Practice, Tehran, Iran

**Keywords:** Cervical Vertebrae, Dental Occlusion, Jaw Relation Record

## Abstract

**Background:**

For determining the cervical column curvature, the curve fitting method is the most precise method, but using this method in clinic seems to be difficult if not possible. In this study, we used a modification of cervical column inclination angle that has been already mentioned

**Objectives:**

The aim of this study was to evaluate the posture and curvature of the cervical column introducing a modified constructed angle in order to evaluate the cervical column curvature in a relax position in relation to the jaws sagittal position.

**Patients and Methods:**

The lateral cephalometries of patients with no anomaly were taken in the natural head position. The mean age of the patients was 13.49 years including 56 female and 44 male. Steiner and Wits analysis was used to evaluate the sagittal position of the jaws. Modified constructed CVT/HOR and OPT/HOR angles were used to evaluate the cervical column posture and curvature. Patients were classified into three groups according to the angle’s classification.

**Results:**

The results showed a significant positive correlation between modified constructed angles and sagittal jaw relationships (P < 0.05). Besides, in class II patients, there was a significant correlation between OPT/HOR and parameters ANB and Wits (P < 0.05 and P < 0.01, respectively). Age could not affect the curvature and posture of the cervical column.

**Conclusions:**

According to the result of this study using modified constructed angles may be a simple method for evaluation of the relation between cervical column curvature and sagittal position of the jaws. There is significant correlation between cervical column posture angles and parameters ANB and Wits in Cl. II patients.

## 1. Background

The cervical column in a functional and morphological aspect has a close relation with dentofacial structures [1][2]. One of these structures is facial morphology. It has been demonstrated that the cranio-cervical posture is related to the skeletal development of the face, this relationship has not been seen in the cranio-vertical posture [3]. This association in patients with a short brachycephalic face is usually a backwardly inclined cervical column and in patients with a long dolichocephalic face is commonly a decreased cervical column curvature [4][5]. It has been hypothesized that this difference between the cranio-cervical and cranio-vertical posture is due to a different developmental origin for the upper and lower segments of the cervical column. While the development of the upper segment is considered to be closely related to facial development, the lower segment is classically considered as the final upper part of the column [6].

Another structure that has an impact on facial morphology is the three dimensional relations of the jaws. These relations have also been proposed to be affected by the cranio-cervical curvature too. Solow et al. have determined the head inclination by using angles formed between the nasion-sella turcica line, the palatal line and the true vertical line, in which they showed the low correlation between head inclination and craniofacial morphology [5][7]. Festa et al. also have studied the association between mandible length and cervical inclination using lateral cephalometry and have reported a positive correlation between these parameters [8]. D’Attilio et al. have shown the correlation between cervical curvature and mandible length, mandible divergence and overjet (a measure of how far the maxillary incisor teeth are ahead of the mandibular incisors) [9]. Although Michelotti et al. reported that there is some evidence about the correlation between jaw position and cervical inclination, this correlation is low for the lower cervical vertebrae [2]. On the other hand, there are some studies that have rejected any particular correlation in patients with different sagittal jaw postures and cervical column curvatures [10][11]. Tecco et al. showed that there is no correlation between cervical curvature and age, sex, ethnicity and height [12].

In orthodontics, the skeletal sagittal relations of the maxilla and mandible bases have been proposed as a valuable factor for diagnoses and treatment plans. Facial profile of the patients may be well explained by the anterior-posterior relations of the jaws regarding the cranial base. Using a parameter of the relative relation of the jaws will be valuable while considering correlation of both jaws simultaneously with another factor such as the cervical column curvature. This would enhance the treatment prognosis. To make these assessments possible, in several studies the lateral cephalometric radiography has been used for analysis of the head and neck posture [1][4][7][8][9][13][14]. Inclination of the cervical column in several studies has been measured as an angle like Odontoid Process Tangent (OPT)/Horizontal (HOR) and Cervical Vertebra Tangent (CVT)/Horizontal (HOR) [4][5][7][15][16] and the curvature of the cervical column was measured using a curve fitting method [12].

For determining the cervical column curvature, the curve fitting method is the most precise method, but using this method in clinic seems to be difficult if not possible. In this study, we used a modification of cervical column inclination angles that has been already mentioned [4][5][7][15][16]. The advantage of this modified angle for determining the cervical column curvature is its easiness of measurement in a Natural Head Position (NHP) based lateral cephalometry. So if we can easily assess the cervical column curvature with other cephalometric factors with a reliable measure in clinic, the prognosis of treatment can be better prognosticated.

## 2. Objectives

The aim of this study was to determine any correlation between cervical column curvature and sagittal jaw relationships.

## 3. Patients and Methods

### 3.1. Patients

This was a cross sectional study on one hundred pre-treatment lateral cephalograms of the patients (56 females, 44 males; mean age, 13.49 years; SD, 5.53 years) with different skeletal Angle classifications (a type of classification of the occlusion according to the maxillary and mandibular molars and canines into three major groups: Cl.I, Cl.II and Cl.III), who came to the Orthodontics Department of Dental School of Tehran University of Medical Sciences in the last four years. They were selected from the archive. The initial sample consisted of 540 patients. The inclusion criteria were the quality of the cephalograms and the position in which the cephalograms were taken; in this study we used NHP. The exclusion criteria were any anomaly, airway obstruction, Temporo-Mandibular Joint (TMJ) disorders, muscular disorders, orthodontic treatment, systemic or congenital disease and the patients’ visual problems leading to incorrect cephalometric values.

### 3.2. Cephalometric Analysis

All cephalograms were made upon a standardized lateral radiograph (18 × 24 cm film, Kodak, Germany) on the basis of NHP (Patient midplane-X ray source distance 146 cm; Patient midplane-film distance 13.5 cm; enlargement factor 1%; exposure: 4-21 mAs, 60-80 kV) by a single technician in the Radiology Department of Dental School of Tehran University of Medical Sciences.

The method used for making radiographs on the basis of NHP, was the most practical method according to the Solow theory in 1971 [17]. Therefore, the true vertical line in the cephalogram was determined and the true horizontal was easily drawn by making a perpendicular line on the true vertical. At last, the total procedure is accomplished in 1 to 3 minutes.

### 3.3. Cephalometric Measurements

The parameters used in tracing are given in Table 1. The ANB (a cephalometric angular measurement of the anterior-posterior relationship of the maxilla with the mandible) angle and Wits measure (a cephalometric linear measurement of the anterior-posterior relationship of the maxilla with the mandible) were used respectively according to Steiner and Wits analysis in order to classify cases to Angle’s malocclusion classes [18][19].

**Table 1:** Parameters Used in This Study

	**Definition**
Age	The age of the patient in year
ANB	Stainer analysis in degree
Wits	Wits analysis in millimeter
OPT a/HOR a	OPT angle with true horizontal in degree
CVT a/HOR	CVT angle with true horizontal in degree
SNGoGn	SNGoGn angle in degree
Class	Angle classification (Cl.I, Cl.II, Cl.III)
MCA a	The angle between OPT/HOR and CVT/HOR lines in degree
Phi	The angle of the tangent line with true horizontal line in point CV2IP in degree

^a^ Abbreviations: CVT, Cervical vertebra tangent; HOR, Horizontal; MCA,Modified cervical angle; OPT, Odontoid process tangent

### 3.4. Measurement of Cervical Curvature

For determining the cranio-cervical posture, the points and lines used were according to the Solow and Tallgren studies. Odontoid Process Tangent (OPT) is the posterior tangent line to the odontoid process that passes CV2SP and CV2IP. Cervical Vertebra Tangent (CVT) is the posterior tangent line to the odontoid process that passes CV2SP and CV4IP.

**Figure 1:**
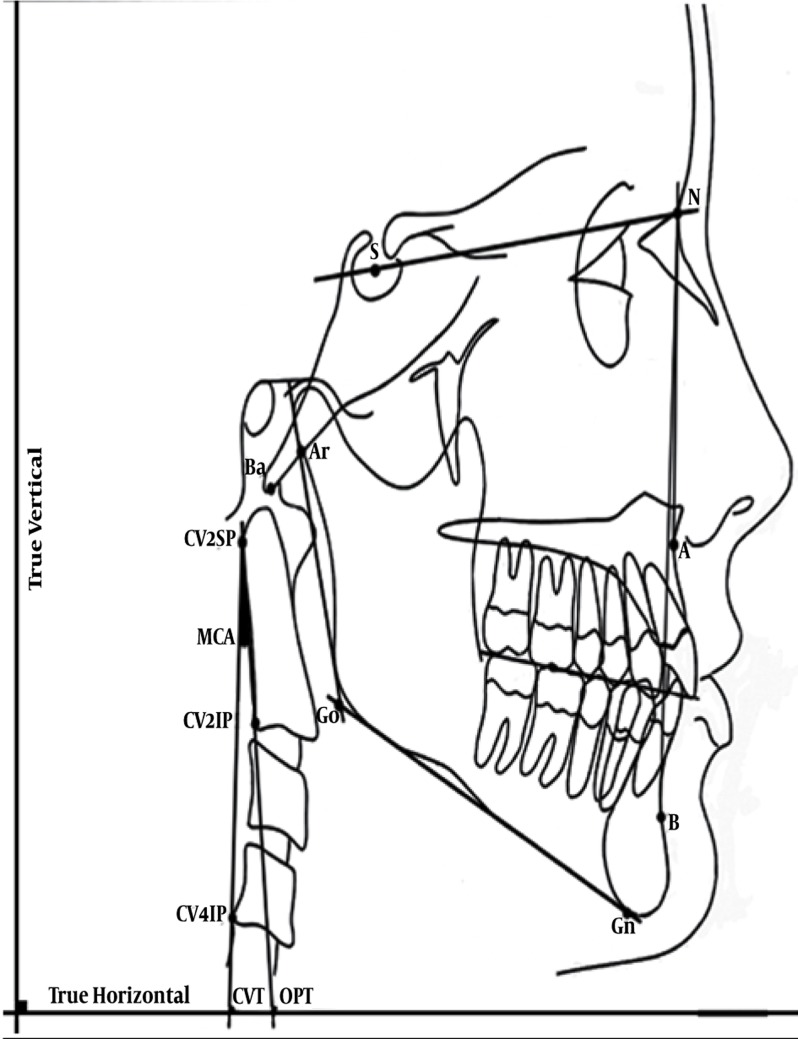
The Illustration Shows Schematic Method of a Traced Cephalometry. S, Sella turcica; N, Nasion; Gn, Gnathion; Go, Gonion; Ar, Articular; Ba, Basion; CV2SP, The most superior posterior point of CV2; CV2IP, The most inferior posterior point of CV2; CV4IP, The most inferior posterior point of CV4; CVT, The posterior tangent line to odontoid process that passes CV2SP and CV4IP; OPT, The posterior tangent line to odontoid process that passes CV2SP and CV2IP

CV2SP is the most superior and posterior point on the body of the second vertebra. CV2IP and CV4IP is the most inferior and posterior point on the body of the second and fourth vertebra, respectively. The lines that pass CV2SP-CV2IP and CV2SP-CV4IP make angles with the true horizontal (OPT/HOR and CVT/HOR, respectively). The cervical inclination is expressed by angles OPT/HOR and CVT/HOR. The modified cervical angle that defines the degree of the curvature was measured by calculating the difference between OPT/HOR and CVT/HOR angles, named MCA (Modified Cervical Angle). An example of tracing is shown in Figure 1.

**Figure 2:**
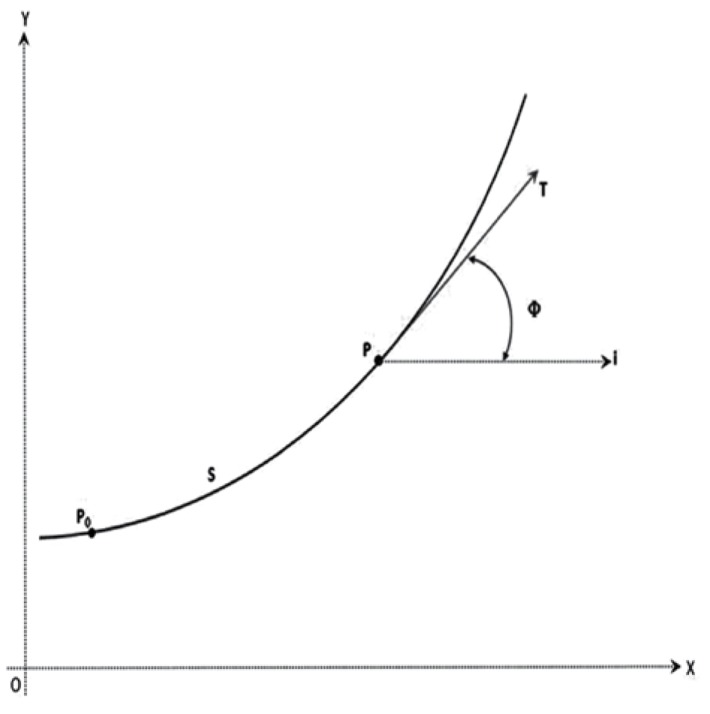
The Gragh Shows the Method of Phi (Ф) Angle Calculation. P0, The beginning point of the path; S, The length of the path; P; The end point of path; T, The tangent line on the curve at point P; Ф, The angle between T and true horizontal

Thomas and Finney describe how to calculate the curvature in every point in a curve [20]. They describe that moving on a derivable curve placed in a plane, when the tangent unit vector (T) bends the curve also turns. The rhythm change of T may be measured by Phi (Ф) angle changes (Figure 2). Phi is the angle between the unit tangent vector (T) and the unit horizontal vector (X) (Figure 2).

In every point like P on the curve, the amount of the curvature named Kappa (К) may be calculated as follows:

**Figure:**
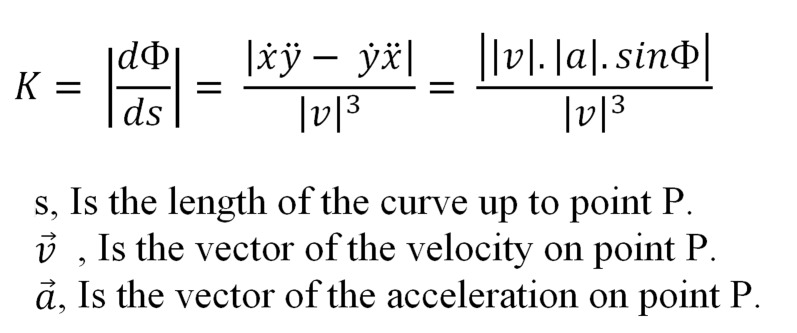


Cervical curvature has been measured by the angle that is formed by two lines, one connects the beginning and the end of the curve points CV2SP and CV4IP, and the other connects the beginning of the curve to point CV2IP that stands between the beginning and the prominent point of the curve. A similar method to this was used by Findlay and Harris and Smith and Van Gisbergen [21].

A positive significant correlation was detected between angle MCA and angle Φ that has been previously shown is directly correlated to the amount of the curvature (κ).

### 3.5. Method Error

The methodological error within the cephalometric analysis was determined by having the seven measurement values analyzed on randomly chosen cephalographs after a one week interval by the same examiner. The error was determined by means of the Dahlberg Formula (mean error ratio SE = *d *^2^/2n, where d = difference between the measurements at two different times; n = number of measurements).

### 3.6. Statistical Analysis

SPSS 17.0 was used to statistically analyze the measured values. The Pearson correlation coefficient was used for the calculation of correlations. The significance level in all tests was determined as P < 0.05.

## 4. Results

Descriptive statistics for the cephalometric measurements are given in Table 2. The methodic error when evaluating angular measurements on the lateral cephalographs was below 0.5°. This value was acceptable according to the reference Trpkova et al. [22]. The group statistics are shown in Table 3. The results of the correlation coefficient are shown in Table 4.

The correlation between MCA and Phi angle was significant (P < 0.0001). The difference between MCA means in three groups of occlusion were statistically significant (P = 0.007). There was no significant correlation between MCA and age (P > 0.05).

**Table 2:** Summary Statistics for Age, Cervical Column Curvature and Cephalometric Variables of the Examined Subjects (n = 100)

	**Minimum**	**Maximum**	**Mean ± SD**
Age	9.00	28.00	13.49 ± 5.53
ANB	- 5.50	15.00	3.70 ± 3.41
Wits	- 15.00	15.00	-0.98 ± 5.25
OPT a /HOR a	72.00	124.00	94.56 ± 9.87
CVT a/HOR	70.00	130.00	98.94 ± 11.63
MCA a	0.00	20.00	5.78 ± 3.16
Phi	81	92	87.69 ± 3.73

^a^ Abbreviations: Cvt, Cervical vertebra tangent; Hor, Horizontal; MCA, Modified cervical angle; Opt, Odontoid process tangent

**Table 3:** Group Statistics

**Angle Classification**	**Angle**	**Age Group**	**No.**	**Mean ± SD**
Overall				
(Cl.I, Cl.II, Cl.III)				
	OPT c/HOR c			
		1 a	53	95.00 ± 9.14
		2 b	47	94.06 ± 10.71
	CVT c/HOR			
		1	53	99.43 ± 10.65
		2	47	98.38 ± 12.74
	MCA			
		1	53	5.83 ± 3.65
		2	47	5.72 ± 2.52
Cl.I				
	OPT/HOR			
		1	7	97.85 ± 11.52
		2	8	90.50 ± 7.38
	CVT/HOR			
		1	7	100.42 ± 13.99
		2	8	87.62 ± 9.34
	MCA c			
		1	7	3.42 ± 2.43
		2	8	4.12 ± 3.04
Cl.II				
	OPT/HOR			
		1	24	94.29 ± 7.94
		2	20	97.65 ± 13.56
	CVT/HOR			
		1	24	98.58 ± 11.21
		2	20	103.50 ± 15.55
	MCA			
		1	24	6.95 ± 4.31
		2	20	6.35 ± 2.32
Cl.III				
	OPT/HOR			
		1	22	94.86 ± 9.83
		2	19	91.78 ± 7.22
	CVT/HOR			
		1	22	100.04 ± 9.25
		2	19	97.52 ± 6.64
	MCA			
		1	22	5.36 ± 2.71
		2	19	5.73 ± 2.32

^a^ Age Group 1: Age 9-11

^b^ Age Group 2: Age more than 18

^c^ Abbrevations: Cvt, Cervical vertebra tangent; Hor, Horizontal; Mca, Modified cervical angle; Opt, Odontoid process tangent

**Table 4:** The Pearson Correlation Coefficient Between Cranio-Cervical Curvature Angles and Occlusion Classification

	**ANB**				**Wits**			
	**Overall**	**Cl. I**	**Cl. II**	**Cl. III**	**Overall**	**Cl. I**	**Cl. II**	**Cl. III**
OPT c/HOR c	0.189	0.186	0.382 a	- 0.093	0.210 a	0.096	0.441 b	- 0.094
CVT c/HOR	0.233 a	0.254	0.408 b	- 0.004	0.229 a	0.169	0.444 b	- 0.053
MCA c	0.300 b	- 0.166	0.168	0.364 a	0.205 a	- 0.145	0.084	0.199

^a^ P < 0.05

^b^ P < 0.01

^c^ Abbrevations: Cvt, Cervical vertebra tangent; Hor, Horizontal; Mca, Modified cervical angle; Opt, Odontoid process tangent

Overall, there was a significant correlation between sagittal jaw relationship parameters and MCA. Furthermore, in Class II patients, there were significant correlations between OPT/HOR and ANB; and also OPT/HOR and Wits.

## 5. Discussion

In 1984 [3], 1986 [15] and 1998 [16] Solow et al. carried out some studies in order to find the effects of craniocervical changes on factors such as the upper airway, growth, development and malocclusion. Finally, they reported that changes in the craniocervical angle correlated with not only the mandible rotation, but also the general growth direction. They also showed the relationship between craniofacial morphology, craniocervical angles and upper airway deficiency, but they said that this is not strong and may be due to the presence of a general growth control mechanism in craniofacial development. Their results showed that there are only few significant correlations between sagittal, vertical and horizontal anomalies with different positions of the head.

Solow et al. used the craniocervical angles in their studies and the cervical column was not noticed separately. Particularly, they did not attend to the cervical curvature. Festa et al. [8] and D’Attilio et al. [9] in their studies used the CVT/EVT (EVT: a line through the infero-posterior points of C4 and C6) angle as the cervical column curvature angle. D’Attilio et al. reported the correlation between cervical curvature and the overjet; this finding exclusive of the method they used, confirms the result of this study. But as said, they used the sixth cervical vertebra in the calculation of the cervical curvature, which according to the results of Lippold et al., [10] the minimum cervical curvature changes occurs.

Tecco et al. in their study traced vertebrae 4 to 7 and used the curve fitting method for calculating the curvature. They showed that the amount of cervical curvature in people with maxillary deficiency that had protruded incisors, was increased, but the amount of curvature did not have significant correlation with the ANB angle. By ignoring the method of curvature calculation, this finding is in contrast with the results of this study [12].

The method that has been used in this study for calculating the curvature defined an angle named MCA. This angle is formed between two cervical lines OPT/HOR and CVT/HOR. As shown, this angle has close relation with cervical curvature changes and C6 vertebra tracing is not necessary. It is also easily measurable in clinic. It was repeatable and the method error in determining the cervical curvature was not significant. So we used it as a cervical curvature indicator. This study showed that in sagittal relations, there is significant positive correlation between MCA and parameters ANB and Wits. As regard to the maximum mean of angle MCA that was in class I patients, we can conclude that whenever the skeletal relations tend to class II, the cervical curvature increases.

Mertensmeier and Diedrich in 1992 showed that patients with class II malocclusion had increased lordosis in the spinal column compared to patients with class III malocclusion. This finding confirms the result of this study, although they did not provide particular results about the cervical column [23]. Results of this research showed the positive significant correlation between cervicohorizontal angles and parameters ANB and Wits in class II patients. In other words, in patients with class II malocclusion, when cervical inclination increases, ANB and Wits parameters also increase. This finding is congruent with Solow et al.’s study in 1998 [3].

In addition, the results showed influence of gender on the cervical column curvature. This influence was also shown in 1987 by Hellsing et al. [24] which was rejected by Borden et al., Retchman et al. and Tecco et al. [12][25][26].

Nevertheless, based on the type of this study that was cross sectional, it is impossible to make any specific conclusion that results in determining etiology or cause and effect relation in parameters. So it is suggested to study these parameters in a longitudinal study.
